# Maternal Serum Thrombospondin-4 Levels in Gestational Hypertension and Risk of Preeclampsia

**DOI:** 10.3390/jcm14207414

**Published:** 2025-10-20

**Authors:** Özgür Volkan Akbulut, Ahmet Arif Filiz, Belgin Savran Üçok, Mustafa Bağcı, Zehra Vural Yılmaz

**Affiliations:** 1 Department of Perinatology, Etlik City Hospital, Ankara 06170, Turkey; ahmetarif35.filiz@gmail.com (A.A.F.); zehravural@gmail.com (Z.V.Y.); 2 Department of Obstetrics and Gynecology, Etlik City Hospital, Ankara 06170, Turkey; dr.belgin@gmail.com; 3 Department of Perinatology, Van Regional Training and Research Hospital, Van 65300, Turkey; mustafabagci@outlook.com.tr

**Keywords:** gestational hypertension, preeclampsia, thrombospondin-4 (TSP-4)

## Abstract

**Background:** Gestational hypertension (GHT) is associated with adverse maternal and perinatal outcomes, and reliable biomarkers for risk stratification remain limited. Thrombospondin-4 (TSP-4), a matricellular glycoprotein implicated in vascular remodeling, may play a role in hypertensive disorders of pregnancy. This study aimed to investigate maternal serum TSP-4 levels in GHT and their associations with obstetric and neonatal outcomes. **Methods:** This prospective cohort study included 44 women with GHT and 44 normotensive controls. Maternal serum TSP-4 levels were measured between 20–30 weeks’ gestation, and demographic, obstetric, and neonatal data were recorded. The development of preeclampsia (PE) and composite adverse perinatal outcomes (CAPO) was subsequently compared between the groups. **Results:** TSP-4 levels were significantly higher in the GHT group compared with controls (9.50 vs. 7.92 ng/mL, *p* < 0.001). Women with GHT had lower gestational age at delivery and birth weight, with higher rates of preterm delivery, fetal distress, NICU admission, and composite adverse perinatal outcomes (all *p* < 0.01). Within the GHT group, patients who developed PE had notably higher TSP-4 levels (13.45 vs. 9.33 ng/mL, *p* < 0.001). **Conclusions:** Elevated maternal serum TSP-4 is independently associated with GHT and progression to PE. TSP-4 may serve as a novel biomarker for risk assessment in hypertensive disorders of pregnancy.

## 1. Introduction

Gestational hypertension (GHT) is defined as the new onset of hypertension after the 20th week of gestation and affects approximately 6% of pregnancies, representing a major cause of maternal and fetal morbidity and mortality [1,2,3]. More severe forms of this disorder include preeclampsia (PE) and HELLP syndrome, with nearly 50% of women diagnosed with GHT eventually progressing to these conditions [4,5]. The underlying pathophysiology of hypertensive disorders of pregnancy is multifactorial and remains incompletely understood. However, hemodynamic alterations play a pivotal role in determining the clinical severity of the disease.

In normal pregnancy, spiral uterine arteries undergo physiological remodeling early in the second trimester, facilitated by extravillous trophoblast invasion. This transformation ensures a low-resistance, high-flow uteroplacental circulation. In contrast, inadequate trophoblast invasion and failure of spiral artery remodeling in GHT and PE result in chronic uteroplacental ischemia [6]. The ischemic environment disrupts the structural and functional integrity of the placenta and triggers the release of numerous bioactive mediators into the maternal circulation, ultimately leading to widespread endothelial injury. These factors include anti-angiogenic molecules, inflammatory microparticles, cell-free nucleic acids, oxidized lipids, and reactive oxygen species [7].

Thrombospondins are matricellular proteins that modulate endothelial function and vascular remodeling under ischemic and inflammatory conditions [8]. Among them, thrombospondin-4 (TSP-4) has more recently been identified as a regulator of trophoblast function and placental vascular development. An experimental study demonstrated decreased TSP-4 expression in placental tissue from women with PE compared with normal pregnancies, predominantly localized in trophoblast cells. Suppression of TSP-4 expression inhibited the transforming growth factor (TGF)-β1 signaling pathway, reducing trophoblast proliferation, migration, and invasion, while these effects were restored by treatment with a TGF-β1 agonist. These findings suggest that TSP-4 facilitates trophoblast invasion and spiral artery remodeling through TGF-β1-mediated mechanisms, and that its downregulation may contribute to the pathogenesis of PE [9].

Other extracellular matrix (ECM) proteins that contribute to endothelial integrity and inflammatory regulation have long been investigated in relation to placental dysfunction and adverse obstetric outcomes. Among these, fibronectin and decorin represent well-established ECM proteins, and their links to preeclampsia have been relatively well documented [10,11,12]. More recently, ECM components such as syndecans and thrombospondins have also been examined, and they are increasingly recognized for their potential roles in the pathogenesis of pregnancy complications [13,14]. Taken together, these findings indicate that alterations in ECM proteins, especially TSP-4, may underlie abnormal placental vascular development and contribute to the clinical spectrum of hypertensive disorders of pregnancy.

Based on this biological behavior, it is plausible that TSP-4 may contribute to the pathophysiological pathways of GHT. Therefore, in the present study, we aimed to investigate maternal serum TSP-4 levels in gestational hypertension compared to normotensive pregnancies and to analyze their associations with obstetric and neonatal outcomes.

## 2. Materials and Methods

### 2.1. Participant Selection

This prospective cohort study was conducted at the Perinatology Department of Ankara Etlik City Hospital between 1 April 2025 and 1 September 2025. The study was conducted in accordance with the Declaration of Helsinki and approved by the Ethics Committee of Ankara Etlik City Hospital on 26 March 2025 (approval number: AESH-BADEK-2025-0415). Written informed consent was obtained from all participants.

A total of 88 pregnant women with singleton pregnancies were enrolled: 44 diagnosed with GHT and 44 normotensive controls matched for gestational age. The diagnosis of GHT was made according to the criteria of the American College of Obstetricians and Gynecologists (ACOG), defined as blood pressure ≥140/90 mmHg on repeated measurements after 20 weeks of gestation in the absence of organ dysfunction or laboratory findings consistent with preeclampsia (PE) [3]. Blood pressure measurements were performed by trained antenatal clinic nurses, following standardized recommendations. Measurements were obtained with participants in the semi-supine position, with the right arm supported at heart level, after a period of rest, and using the same validated digital device. For accuracy, two measurements were obtained at short intervals, and their mean value was considered in the analysis.

Exclusion criteria included pre-existing chronic hypertension and other comorbidities, autoimmune disorders, long-term medication use, a history of smoking or alcohol use, and the presence of PE at the time of blood sampling. In addition, pregnancies conceived by assisted reproductive techniques, multiple gestations, cases with structural or chromosomal fetal anomalies, women whose antenatal follow-ups or deliveries occurred at another center, and those unwilling to participate were also excluded.

All participants were prospectively monitored for the development of preeclampsia through regular blood pressure measurements and laboratory evaluation during antenatal follow-up visits. Maternal demographic and obstetric characteristics, as well as neonatal outcomes including gestational age at delivery, birth weight, Apgar scores, and neonatal intensive care unit (NICU) admission, were recorded. The development of PE and composite adverse perinatal outcomes (CAPO) were also assessed.

### 2.2. Blood Sampling and Laboratory Analysis

Venous blood samples were collected from the antecubital vein between 20 and 30 weeks of gestation. Venous blood samples were collected from patients immediately after the diagnosis of gestational hypertension according to ACOG criteria and before the initiation of medical treatment [3]. All patients were initiated on appropriate antihypertensive therapy after the blood sample was collected. For the control group, venous blood samples were collected from pregnant women with normal blood pressure, no comorbidities, and singleton pregnancies. Pregnant women who underwent blood sampling were followed until the end of pregnancy, and only those who did not develop elevated blood pressure or pregnancy complications until delivery were included in the study. Samples were allowed to clot for 2 h at room temperature or overnight at 2–8 °C, and centrifuged at 2000× *g* for 15 min at 2–8 °C. The resulting serum was carefully separated, aliquoted, and stored at −80 °C until analysis, avoiding repeated freeze–thaw cycles.

Maternal serum TSP-4 levels were measured using a commercially available enzyme-linked immunosorbent assay (ELISA) kit (EasyPro Human TSP-4 ELISA Kit, Feiyuebio, Shanghai, China; Cat No: FY-EH2021S). The assay is based on a double-antibody sandwich principle and provides a detection range of 0.16–10 ng/mL, with a sensitivity of 0.1 ng/mL. According to the manufacturer, no significant cross-reactivity or interference was observed, and the intra- and inter-assay coefficients of variation were <10%. All samples were tested in duplicate to ensure reproducibility. The absorbance was measured at 450 nm using a calibrated microplate reader, and the final concentrations of TSP-4 were calculated based on the standard curve and corrected for the dilution factor, as per the kit instructions. Results were reported in nanograms per milliliter (ng/mL).

### 2.3. Statistical Analysis

Statistical analysis was performed using SPSS software version 22.0 (IBM Corporation, Armonk, NY, USA). The Kolmogorov–Smirnov test was used to assess the normality of distributions. Descriptive statistics of continuous variables were expressed as mean ± standard deviation for normally distributed data and median (min–max) for non-normally distributed data. Categorical variables were compared using the chi-square test or Fisher’s exact test. Continuous variables were compared using the independent-samples *t*-test for normally distributed data and the Mann–Whitney U test for non-normally distributed data. Receiver operating characteristic (ROC) curve analysis was used to calculate and compare the area under the curve (AUC) and to determine optimal cutoff values according to the Youden index. A *p*-value < 0.05 was considered statistically significant.

## 3. Results

The comparison of demographic, clinical, and perinatal characteristics between groups is presented in Table 1. A total of 44 pregnant women with GHT and 44 healthy controls were included. Maternal age, gravida, parity, abortus, BMI, and gestational age (GA) at blood sampling were comparable between groups (*p* > 0.05). Serum TSP-4 levels were significantly higher in the GHT group (9.50 [8.28–10.00] ng/mL) compared to controls (7.92 [6.07–8.97] ng/mL; *p* < 0.001). GA at birth and birth weight were significantly lower in the GHT group (34.9 [31.7–37.3] weeks vs. 38.6 [37.5–39.2] weeks, *p* < 0.001; 2215 ± 821 g vs. 3179 ± 436 g, *p* < 0.001). Adverse perinatal outcomes were more frequent in the GHT group, including 5th-min Apgar score ≤ 7 (25.0% vs. 9.1%, *p* = 0.002), preterm birth (56.8% vs. 0%, *p* < 0.001), fetal distress (15.9% vs. 0%, *p* = 0.012), NICU admission (40.9% vs. 11.4%, *p* = 0.002), and CAPO (56.8% vs. 11.4%, *p* < 0.001). In the study group, PE developed in 9 patients. TSP-4 levels were significantly higher in these patients (13.45 [11.75–16.12] ng/mL) compared to those with GHT alone (9.33 [7.96–9.89] ng/mL; *p* < 0.001). The Mann–Whitney U test revealed a significant difference in TSP-4 levels between the hypertensive and healthy groups (Z = −4.394, *p* < 0.001). The effect size, expressed as the rank-biserial correlation, was r = 0.544 (95% CI: 0.351–0.692), indicating a large effect.

The comparison of maternal and perinatal characteristics between CAPO and non-CAPO groups is presented in Table 2. Among GHT patients, 25 (56.8%) developed CAPO. Maternal age, gravida, parity, abortus, BMI, GA at blood sampling, and TSP-4 levels did not differ significantly between CAPO and non-CAPO groups (*p* > 0.05). However, GA at birth was significantly lower in the CAPO group (33 [30–34] weeks vs. 38 [37,38] weeks; *p* < 0.001), and birth weight was significantly reduced (1695 ± 604 g vs. 2898 ± 507 g; *p* < 0.001).

TSP-4 levels demonstrated a significant positive correlation with the presence of GHT (r = 0.471, *p* < 0.001) and with the development of PE among GHT patients (r = 0.497, *p* < 0.001). No significant correlations were observed between TSP-4 levels and maternal age, GA at blood sampling, BMI, GA at birth, or birth weight in the control group (*p* > 0.05). These correlation analyses are presented in Table 3.

**Figure 1:**
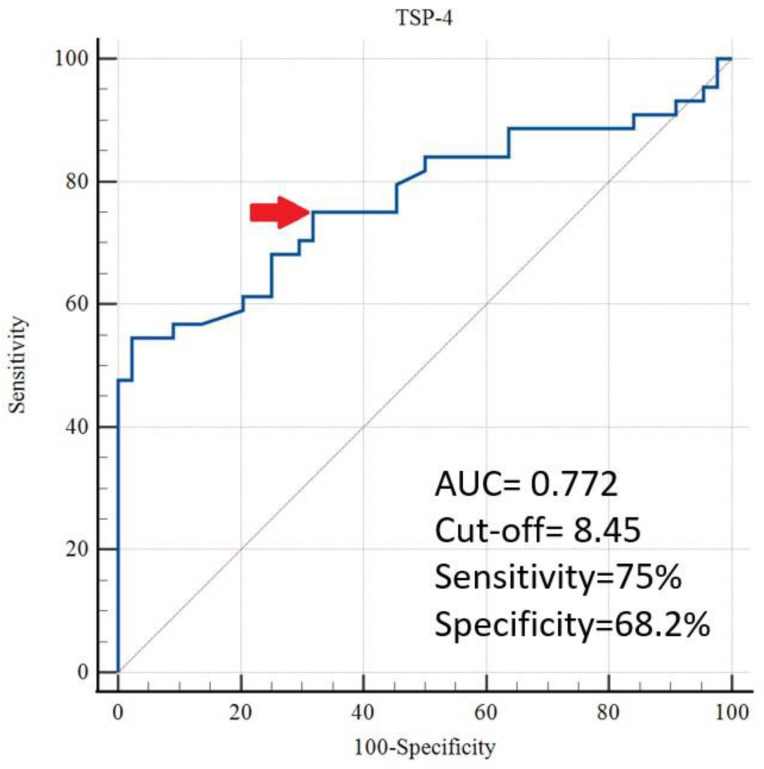
Evaluation of TSP-4 for the prediction of gestational hypertension by ROC analysis (The cut-off value is indicated by an arrow).

Table 4 shows that the ROC analysis identified a TSP-4 cut-off value of >8.45 ng/mL for predicting GHT, with an area under the curve (AUC) of 0.772 (95% CI, 0.670–0.855), a sensitivity of 75.0%, a specificity of 68.2%, a positive likelihood ratio (+LR) of 2.36, a negative likelihood ratio (−LR) of 0.37, and a *p* < 0.001 (Figure 1).

In multivariable logistic regression analysis, after adjusting for maternal age, gravida, gestational age at blood sampling, and BMI, TSP-4 levels were independently associated with gestational hypertension (aOR 1.526; 95% CI, 1.171–1.987; *p* = 0.002) (Table 5).

## 4. Discussion

In this study, maternal serum levels of TSP-4 were significantly elevated in pregnant women with GHT compared to normotensive controls. Moreover, TSP-4 was independently associated with the presence of GHT and progression to preeclampsia (PE). These findings suggest that TSP-4 may play a role in the pathophysiology of hypertensive disorders of pregnancy and has potential as a biomarker for disease stratification.

Thrombospondins are matricellular glycoproteins involved in tissue remodeling, angiogenesis, and inflammation. Among these, TSP-4 is associated with cardiovascular remodeling, endothelial dysfunction, and fibrosis—processes that play a crucial role in the pathogenesis of hypertensive disorders of pregnancy [15,16]. Our finding that TSP-4 levels are significantly higher in GHT patients is consistent with previous studies showing that TSP-4 expression is upregulated in patients with gestational hypertensive disorders [9]. Previous studies have demonstrated that TSP-4 may influence several molecular pathways involved in angiogenesis and vascular remodeling. By modulating the transforming growth factor (TGF)-β1 signaling cascade, TSP-4 regulates trophoblast proliferation, migration, and invasion, thereby contributing to spiral artery remodeling and placental vascular development. Beyond its role in the TGF-β1 pathway, TSP-4 also interacts with the vascular endothelial growth factor (VEGF) and placental growth factor (PlGF) axis, both of which are central mediators of angiogenic balance during pregnancy. Disruption of this delicate VEGF/PlGF equilibrium is a hallmark of hypertensive disorders of pregnancy and leads to endothelial dysfunction and impaired placental perfusion. Elevated TSP-4 expression may further aggravate this imbalance by promoting extracellular matrix deposition, reducing nitric oxide bioavailability, and increasing vascular stiffness, all of which can exacerbate ischemic and inflammatory injury within the placental circulation [9,17]. Collectively, these mechanisms provide a biologically plausible framework linking TSP-4 to the pathogenesis of gestational hypertension and support its potential utility as a biomarker reflecting endothelial and vascular maladaptation in pregnancy.

The pathophysiology of GHT is multifactorial and not fully understood, but endothelial dysfunction, placental hypoperfusion, and exaggerated maternal inflammatory responses play a central role [18,19]. TSP-4 has been shown to enhance endothelial cell migration, alter extracellular matrix organization, and promote the production of inflammatory cytokines. Therefore, elevated TSP-4 levels could reflect vascular and placental maladaptation in GHT and serve as a surrogate marker for disease activity [20].

Remarkably, we also found significantly higher TSP-4 levels in patients who developed PE compared to those with GHT alone. Progression from GHT to PE is associated with increased systemic inflammation, oxidative stress, and anti-angiogenic imbalance, particularly involving vascular endothelial growth factor (VEGF) and placental growth factor (PlGF) [21,22]. TSP-4 could act synergistically in this context by enhancing vascular injury and maladaptive remodeling. These findings support its potential as an early biomarker to identify GHT patients at high risk of progression to PE.

Although maternal TSP-4 levels were higher in the gestational hypertension group, no significant association was found between TSP-4 concentrations and composite adverse perinatal outcomes. This lack of correlation may be explained by the multifactorial nature of perinatal complications, which are influenced not only by maternal biochemical status but also by factors such as gestational age at delivery, fetal condition, and the quality and timing of clinical management. Prompt obstetric interventions and optimized perinatal care may have mitigated the potential adverse effects of elevated TSP-4 levels on neonatal outcomes. Moreover, the relatively limited sample size in our study may have reduced the statistical power to detect subtle associations. Future studies with larger populations and longitudinal designs are warranted to clarify whether TSP-4 could serve as a reliable biomarker for predicting neonatal prognosis in hypertensive pregnancies.

According to a study by Velrohren et al., the sFlt-1/PlGF ratio was significantly higher in the GHT group than in the control group in pregnancies over 34 weeks. However, this increase was not observed in pregnancies under 34 weeks [23]. The sFlt-1/PlGF ratio reflects the anti-angiogenic imbalance, which is more pronounced in PE cases than in GHT. Therefore, TSP-4 appears more promising for predicting GHT than the sFlt-1/PlGF ratio. The ROC analysis showed that TSP-4 had an AUC of 0.772 in predicting GHT, with 75% sensitivity and 68.2% specificity, indicating moderate diagnostic performance. Although promising on its own, its clinical utility may be enhanced when used in combination with other angiogenic or inflammatory markers such as sFlt-1, PlGF, or C-reactive protein, which have been shown to improve predictive performance when assessed together [24,25]. These findings suggest that TSP-4 could complement existing diagnostic tools, particularly in resource-limited settings where access to more costly assays is restricted.

This study has several limitations, including the modest sample size, single-center design, and single time-point measurement of TSP-4, which precludes evaluation of longitudinal changes. Nevertheless, to our knowledge, this is among the first studies to investigate TSP-4 in GHT and its association with the risk of progression to PE. Larger multicenter prospective studies with serial sampling are needed to confirm these findings and to further elucidate the mechanistic role of TSP-4 in hypertensive disorders of pregnancy.

## 5. Conclusions

Maternal serum TSP-4 levels are elevated in GHT and independently associated with progression to PE, highlighting its potential as a novel biomarker in hypertensive disorders of pregnancy. Looking ahead, future research should focus on integrating TSP-4 into a multi-marker screening framework that combines angiogenic, inflammatory, and endothelial biomarkers such as sFlt-1, PlGF, and C-reactive protein to enhance early prediction and risk stratification. Additionally, the relationship between TSP-4 and gestational hypertension should be investigated in more detail by expanding the study population to include groups with essential hypertension and preeclampsia, and by conducting subgroup analyses. Longitudinal cohort studies with serial measurements at different gestational stages would provide valuable insights into the temporal dynamics of TSP-4 and its interactions with other molecular pathways. Such studies could clarify whether TSP-4 serves only as a disease indicator or plays an active role in the underlying pathophysiological cascade, ultimately paving the way for targeted preventive or therapeutic interventions in high-risk pregnancies.

## Figures and Tables

**Table 1:** Comparison of Maternal Characteristics, Perinatal Outcomes and Serum Thrombospondin-4 Levels in Between Study Groups.

Variables	GHT (n = 44) (50%)	Control (n = 44) (50%)	*p*-Value
Demographic and Laboratory Characteristics			
Maternal age	27.8 ± 4.6	27.9 ± 5.1	0.862 ^a^
Gravida	2 (2–3)	2 (1–3)	0.365 ^b^
Parity	1 (0–2)	1 (0–1)	0.121 ^b^
Abortus	0 (0–1)	0 (0–1)	0.898 ^b^
BMI	29.20 ± 3.98	28.14 ± 5.07	0.275 ^a^
Gestational age at blood sampling	24.3 ± 1.4	24.9 ± 1.9	0.083 ^a^
TSP-4	9.50 (8.28–10.00)	7.92 (6.07–8.97)	<0.001 ^b^
Gestational age at birth	34.9 (31.7–37.3)	38.6 (37.5–39.2)	<0.001 ^b^
Perinatal Outcomes			
Birth Weight (gr)	2215 ± 821	3179 ± 436	<0.001 ^a^
5th minute APGAR score ≤7	11 (25.0%)	4 (9.1%)	0.002 ^c^
Preterm birth	25 (56.8%)	0 (0%)	<0.001 ^c^
Fetal distress	7 (15.9%)	0 (0%)	0.012 ^c^
NICU admission	18 (40.9%)	5 (11.4%)	0.002 ^d^
CAPO	25 (56.8%)	5 (11.4%)	<0.001 ^d^
Variables	PE (n = 9)	GHT (n = 35)	*p*-value
TSP-4	13.45 (11.75–16.12)	9.33 (7.96–9.89)	<0.001 ^b^

GHT: Gestational hypertension, PE: Preeclampsia, BMI: Body mass index, TSP-4: Trombospondine-4, NICU: Neonatal intensive care unit, CAPO: Composite adverse perinatal outcome, ^a^: Student *t*-test, ^b^: Mann–Whitney U, ^c^: Fisher’s exact test, and ^d^: Chi-square.

**Table 2:** Comparison of Maternal and Perinatal Characteristics Between Gestational Hypertension Patients with and without CAPO.

Variables	CAPO (n = 25) (56.8%)	Non-CAPO (n = 19) (43.2%)	*p*-Value
Maternal age	27.8 ± 4.7	27.7 ± 4.7	0.935 ^a^
Gravida	2 (2–3)	2 (1–4)	0.845 ^b^
Parity	1 (0–2)	1 (0–2)	0.264 ^b^
Abortus	0 (0–1)	0 (0–1)	0.459 ^b^
BMI	29.16 ± 3.69	29.26 ± 4.43	0.933 ^a^
Gestational age at blood sampling	23.7 ± 1.5	23.7 ± 1.4	0.951 ^a^
TSP-4	9.46 (8.56–11.75)	9.53 (8.00–9.90)	0.538 ^b^
Gestational age at birth	33 (30–34)	38 (37–38)	<0.001 ^b^
Birth weight (gr)	1695 ± 604	2898 ± 507	<0.001 ^a^

CAPO: Composite adverse perinatal outcome, BMI: Body mass index, TSP-4: Trombospondine-4, ^a^: Student *t*-test, and ^b^: Mann–Whitney U.

**Table 3:** Correlation of Maternal Serum Thrombospondin-4 Levels with Gestational Hypertension, Preeclampsia Development and Clinical Parameters.

Variables	r	*p*-Value
GHT (for all patients)	0.471	<0.001
Development of Preeclampsia (among GHT patients)	0.497	<0.001
Age (years) (among control group)	0.068	0.659
Gestational age at blood sampling (week) (among control group)	0.030	0.847
BMI (kg/m^2^) (among control group)	0.039	0.804
Gestational age at Birth (week) (among control group)	0.264	0.083
Birth Weight (gr) (among control group)	0.225	0.142

GHT: Gestational hypertension and BMI: Body mass index.

**Table 4:** Receiver Operating Characteristic (ROC) Analysis of Serum Thrombospondin-4 Levels for Predicting Gestational Hypertension.

	LR+	LR−	Cut-Off *	Sensitivity	Specificity	AUC	%95 CI	*p*-Value
TSP-4	2.36	0.37	>8.45	75%	68.2%	0.772	0.670–0.855	<0.001

* Cut-off values were found according to Youden index. TSP-4: Trombospondine-4, LR+: Positive likelihood ratio, LR−: Negative likelihood ratio, AUC: Area under the curve, and CI: Confidence Interval.

**Table 5:** Multivariable Logistic Regression Analysis of Serum Thrombospondin-4 Levels in Relation to Gestational Hypertension.

Variables	aOR	95% CI	*p*-Value
Gestational Hypertension			
TSP-4	1.526	1.171–1.987	0.002

TSP-4: Trombospondine-4, aOR: Adjusted odds ratio, and CI: Confidence Interval.

## Data Availability

Due to hospital policies, patient data and study materials cannot be shared. However, the data are available from the corresponding author upon reasonable request.
